# Reassessment Individual Growth Analysis of the Gulf Corvina, *Cynoscion othonopterus* (Teleostei: Sciaenidae), Using Observed Residual Error

**DOI:** 10.3390/ani15142008

**Published:** 2025-07-08

**Authors:** Eugenio Alberto Aragón-Noriega, José Adán Félix-Ortiz, Jaime Edzael Mendivil-Mendoza, Gilberto Genaro Ortega-Lizárraga, Marcelo Vidal Curiel-Bernal

**Affiliations:** 1 Unidad Guaymas del Centro de Investigaciones Biológicas del Noroeste, Km 2.35 Camino al Tular, Estero de Bacochibampo, Guaymas 85454, Sonora, Mexico; 2 Facultad de Ciencias del Mar, Universidad Autónoma de Sinaloa, Paseo Claussen S/N, Mazatlán 82000, Sinaloa, Mexico; feocabo@uas.edu.mx; 3 Departamento de Ingenierías, Tecnológico Nacional de México, Campus Valle del Yaqui, Bácum 85276, Sonora, Mexico; jmendivil.mendoza@itvy.edu.mx; 4 Instituto Mexicano de Investigación en Pesca y Acuacultura Sustentables, Centro Regional de Investigación Acuícola y Pesquera, Calzada Sábalo-Cerritos s/n, col. Estero El Yugo, Mazatlán 82000, Sinaloa, Mexico; gilberto.ortega@imipas.gob.mx; 5 Instituto Mexicano de Investigación en Pesca y Acuacultura Sustentables, Centro Regional de Investigación Acuícola y Pesquera-Guaymas, Calle 20 No. 605-Sur, Guaymas 85400, Sonora, Mexico

**Keywords:** growth model, Bayesian information criterion, error structure, residual analysis, Upper Gulf of California

## Abstract

Growth is the most influential aspect in demographic species analysis. Collecting data on ages and sizes (such as length and weight) is a fundamental step in growth modeling, particularly in fishery science. Residual analysis plays a crucial role in parameterizing the mathematical models chosen to describe the growth patterns of the species under investigation. Using optimal residual criteria is essential to improving model performance and accuracy. In the present study, the length-at-age data of the Gulf corvina (*Cynoscion othonopterus*) were evaluated with the Schnute model to obtain the best error type and to establish the most accurate growth pattern. Later, the observed, constant, depensatory, and compensatory variance approaches were tested using the logistic model. The Bayesian information criterion (BIC) was used as the goodness-of-fit test to obtain the best variance approach parametrizing the growth model. The BIC values selected the observed variance as the best approach to parametrize the logistic growth model. The conclusion is that the observed variance approach produces robust results—that is, the observed variance produced the most plausible fits. It is suggested that the observed error structure should be used to estimate individual growth.

## Simple Summary

The Gulf corvina (*Cynoscion othonopterus*) is a fish belonging to the Sciaenidae family endemic to the Gulf of California, commonly known as croaker. This species is migratory, annually moving to the Upper Gulf of California and the Colorado River Delta biosphere reserve between February and May to reproduce. It is highly valued in fisheries due to its catch volume and its availability during Lent, a period when Mexicans traditionally consume more fish in place of red meat, following Catholic customs. Therefore, responsible management is essential to ensure sustainable exploitation. Effective management requires an understanding of the species growth patterns, which has led to the development of various mathematical models and analytical approaches. In this study, we use a criterion that is gaining prominence for estimating parameters within mathematical growth equations: observed variance. This criterion is compared to those traditionally used in previous studies aimed at assessing the growth of fish in fisheries and aquaculture.

## 1. Introduction

The Gulf corvina (*Cynoscion othonopterus*) is endemic to the Gulf of California and forms spawning aggregations in the Upper Gulf, where water depths are no more than 30 m. This species is commercially harvested within the biosphere reserve, with annual landings exceeding 4000 tons caught by approximately 700 small-scale boats from February to April. Since 2012, the species has been managed through a total allowable annual catch scheme [1]. The Gulf corvina is estimated to reach maturity at two years [2] and is considered relatively fast-growing. Its great value in economic terms comes from the fact that this fish is commercialized for its flesh, swim bladder, and gonads separately. In open access (de facto or de jure) regimes such as that for the Gulf corvina, which forms spawning aggregations, the economic value of the fish can encourage fishing efforts that lead to stock depletion, until the stock reaches risky levels. In such cases, bioeconomic models can provide useful information for management [3]. These models are based on individual growth analysis, and historically, the most commonly used model is the von Bertalanffy growth model (VBGM). Beverton and Holt [4] were the first to use it for fish stock evaluation. Bioeconomic models play a crucial role in fishery management by integrating ecological dynamics and economic considerations. These models not only help in determining optimal harvest levels but also aid in assessing the risks associated with stock depletion. Bioeconomic models account for stock depletion risks by modeling the interplay between fish population dynamics and fishing efforts. For instance, the Gordon–Schaefer model integrates biological growth rates, carrying capacity, and fishing effort to determine the maximum sustainable yield (MSY).

### 1.1. Growth Models

Besides the VBGM, other models such as the Gompertz and logistic growth models have also been used. Since Katsanevakis [5] published his study on the multi-model approach, most related papers have focused on models rather than growth pattern (e.g., the VBGM projects an inverted exponential curve, while the Gompertz and logistic models project a sigmoidal curve). The growth pattern depends not only on the species but also on the stage of the species (larval, juvenile, or adult), meaning that the anticipated growth pattern might be asymptotic (inverted exponential or sigmoidal) or non-asymptotic (linear, exponential, or power). This distinction is important in modeling and understanding long-term behavior. Asymptotic growth is that which approaches a limiting value (a horizontal asymptote) but never quite reaches it, while non-asymptotic growth does not approach a fixed upper bound. It might be linear, exponential, or of other types. The Schnute model is a very versatile model that may project all of these types of curves under special circumstances [6]. This model is a general four-parameter growth model that can take several mathematical forms depending on the values of its coefficients. It is most important to determine the curve that fits the set of parameters obtained for the species under study. The Schnute model has many special cases depending on the values of the parameters *a* and *b*. It can replicate the VBGM, Gompertz, and logistic models, among others. The advantage of using the Schnute model is that there are no differences in parameter meanings. In other words, there may be different reasonable biological interpretations for each parameter, but as the Schnute equations project the same curves for the sub-models, the meaning of the parameters remains unaltered.

The logistic and Schnute models are autonomous, but using appropriate values for parameters *a* and *b* in the latter can provide a sigmoid asymptotic curve similar to the logistic model.

### 1.2. Analytical Approaches

Growth models, like any other mathematical model, consist of variables and parameters that must be estimated. Parameter estimation also involves several analytical approaches, one of which is the use of objective functions to estimate nonlinear models. Objective functions are constructed using the error structure, and this, in turn, also has several considerations. Additive or multiplicative errors are considered, while objective functions with depensatory [7] and compensatory [8] variances have also been tested. This study presents a new approach to estimate parameters of individual growth models based on error structure as previously proposed [9]. It seems obvious that the observed error structure (sample variance) is suitable for parameter estimation in growth modeling; however, it is not used, and instead, the established paradigm is the use of additive or multiplicative error. Based on the above, one can set up an objective function based on different criteria of the standard deviation structure as follows: constant variance, increasing variance (also called a depensatory effect) [7,10], decreasing variance (also called a compensatory effect) [8], and the observed variance, which does not assume a predefined variance structure, instead utilizing the sample-derived variance estimated from the data.

### 1.3. Research Objective

The focus on variability at age has become a core strategy in growth analysis. The main purpose of this study was to estimate the individual growth of the Gulf corvina (*C. othonopterus*), comparing the previously used hypothesis of variability at age, assumed as constant, depensatory, and compensatory, with the observed variance to parametrize the growth model in Gulf corvina from the Upper Gulf of California. The hypothesis of the study is that using the observed error structure will improve the robustness of individual growth parameter estimates.

## 2. Materials and Methods

### 2.1. Data Source

Data were obtained from the SNIB-CONABIO database maintained by CONABIO (Mexico City, Mexico) (http://www.conabio.gob.mx/institucion/cgi-bin/datos.cgi?Letras=L&Numero=298, accessed on 28 February 2025). Detailed information is presented in the above link for those interested in the number of fish sampled by sex, the number of fish sampled, and any additional details. The data represent the length at age of Gulf corvina from the spawning aggregation area in the Upper Gulf of California. Age was estimated using the sagittal otoliths. The total lengths of 463 individuals (246 males, 217 females) were used to model Gulf corvina growth. Gulf corvina fisheries focus on catching larger fish, including individuals that may exceed the average size at age, while bycatch fisheries tend to capture smaller fish, potentially those below the average size at age [2].

### 2.2. Schnute Model Description

The Schnute model [6] was applied to length-at-age data to identify the growth pattern that best fits the observations and to estimate individual growth parameters. This model is a flexible, four-parameter growth function that can take on four distinct mathematical forms, depending on the values of parameters *a* and *b* relative to zero. Although the Schnute model encompasses multiple solution forms, it remains a single unified model [6]. Specific cases of this model replicate well-known growth models: for example, the von Bertalanffy model corresponds to Schnute case 1 with *a* > 0 and *b* = 1, while the logistic model corresponds to Schnute case 1 with *a* > 0 and *b* = −1. In these special cases, *b* is fixed, reducing the model to a three-parameter form, thus simplifying parameter estimation. In this study, we focused on Schnute case 1 where *a* ≠ 0 and *b* ≠ 0, which is described in detail below:

(1) $$L_{t}=\left.\;\right.{\left\{Y_{1}^{\;b}+\left.\left(\;Y_{2}^{\;b}-Y_{1}^{\;b}\right.\right)\left.\left[\frac{1-e^{-a\left.\left(t-\tau_{1}\right.\right)}}{1-e^{-a\left.\left(\tau_{2}-\tau_{1}\right.\right)}}\;\right.\right]\right\}}^{\frac{1}{b}}$$

The following parameters are used in this model:

*t* is the age at size.

$\tau_{1}$ is the lowest age in the data set.

$\tau_{2}$ is the highest age in the data set.

a is the relative growth rate parameter.

b is the incremental relative growth rate (incremental time constant).

$Y_{1}$ is the size at age $\tau_{1}$.

$Y_{2}$ is the size at age $\tau_{2}$.

To compute *L_∞_* using the Schnute model, the following equation was used:

(2) $$L_{\infty }={\left.\left[\frac{\;e^{a\tau_{2}}{\;Y}_{2}^{\;b}-e^{a\tau_{1}\;}Y_{1}^{\;b}}{e^{a\tau_{2}}-e^{a\tau_{1}}}\;\right.\right]}^{\frac{1}{b}}$$

To compute τ_0_, the following equation was used:

(3) $$\tau_{0}=\tau_{1}+\tau_{2}-\frac{1}{a}ln\left.\left[\frac{\;e^{a\tau_{2}}Y_{2}^{\;b}-e^{a\tau_{1}}Y_{1}^{\;b}}{Y_{2}^{\;b}-Y_{1}^{\;b}}\;\right.\right]$$

To compute $\tau^{*}$, the following equation was used:

(4) $$\tau^{*}=\tau_{1}+\tau_{2}-\frac{1}{a}ln\left.\left[\frac{\;b\left(e^{a\tau_{2}}Y_{2}^{\;b}-e^{a\tau_{1}}Y_{1}^{\;b}\right)}{Y_{2}^{\;b}-Y_{1}^{\;b}}\;\right.\right]$$

To estimate the parameters, the objective functions were first suited considering the following: $Y_{o}$ is the observed value of the dependent variable and $Y_{e}$ is the estimated value with any of the candidate models. The likelihood function was calculated as follows:

(5) $$LL=\sum \left(-0.5ln\left(\sigma^{2}\right)-0.5ln\left(2\pi \right)-\frac{{\left(Y_{o}-Y_{e}\right)}^{2}}{2\sigma^{2}}\right)$$

This function was maximized. The sigma $\left(\sigma \right)$ values used according to error structure criteria were as follows:

Observed:

(6) $$\sigma_{i}=\sqrt{\frac{\sum {\left(Y_{oi}-Y_{ai}\right)}^{2}}{n}}$$

In this case, $Y_{oi}$ is the observed value at each age and $Y_{ai}$ is the average value at each age.

Additive:

(7) $$\sigma =\sqrt{\frac{\sum {\left(Y_{o}-Y_{e}\right)}^{2}}{n}}$$

Multiplicative:

(8) $$\sigma =\sqrt{\frac{\sum {\left({lnY}_{o}-{lnY}_{e}\right)}^{2}}{n}}$$

In this case, $Y_{o}$ is the observed value and $Y_{e}$ is the estimated value.

### 2.3. Logistic Model Description

The logistic model was also analyzed to test the different standard deviation structures. This model anticipates a sigmoid-shaped curve with an inflection point at 50% (symmetrical curve). Reference [8] probed this model to analyze compensatory (decreasing the variance of errors with x, the independent variable) and depensatory (increasing variance of errors with x) variance. The logistic equation is as follows:

(9) $$Y_{t}=Y_{\infty }{\left(1+e^{-k\left(t-t^{*}\right)}\right)}^{-1}$$

where $Y_{t}$ is the size at time t, $Y_{\infty }$ is the asymptotic size, $t^{*}$ is the inflection point of the sigmoid curve, and k represents the coefficient of growth.

A normal distribution of errors was considered (additive error). The sigma $\left(\sigma \right)$ values used according to error structure criteria were as follows:

Constant:

(10) $$\sigma =\sqrt{\frac{\sum \;{\left(Y_{o}-Y_{e}\right)}^{2}}{n}}$$

Depensatory:

(11) $$\sigma =\sqrt{{\sigma_{\infty }^{2}\;\left[{\left(1+e^{-k\left(t-t^{*}\right)}\right)}^{-1}\right]}^{2}}$$

Compensatory:

(12) $$\sigma =\sqrt{{\sigma_{\infty }^{2}\;\left[{\left(1+e^{-k\left(t-t^{*}\right)}\right)}^{-1}\right]}^{-2}}$$

Observed:

(13) $$\sigma_{i}=\sqrt{\frac{\sum {\left(Y_{oi}-Y_{ai}\right)}^{2}}{n}}$$

where $\sigma_{\infty }^{2}\;$ is the variance for the oldest organism, like $\;L_{\infty }$ in growth models. That is, $\sigma_{\infty }^{2}$ is the variance at the asymptotic size. $Y_{o}$ is the observed value, $Y_{e}$ is the estimated value, $Y_{oi}$ is the observed value at each age, and $Y_{ai}$ is the average value at each age.

### 2.4. Model Selection Criterion

The Bayesian information criterion (BIC) was employed to identify the optimal error structure for parameterizing the growth models. The BIC was estimated as $BIC=2-LL+ln\left(n\right)\theta_{i}$, where LL is the maximum log-likelihood, $\theta_{i}$ is the number of parameters, and *n* represents the number of observations. The error structure achieving the lowest BIC value is best for model parameter estimation. Differences in the BIC values $\left(\Delta_{i}={BIC}_{i}-{BIC}_{min}\right)$ were estimated among the error structures used in this study. The BIC weight $w_{i}$ is the percentage of evidence in favor of error structure i. $w_{i}$ was estimated according to Burnham and Anderson [11] using the following formula:

(14) $$W_{i}=\frac{e^{\left(-0.5\Delta_{i}\right)}}{\sum_{i=1}^{3}e^{\left(-0.5\Delta_{i}\right)}}$$

### 2.5. Confidence Intervals

Confidence intervals were assessed using likelihood profiles in combination with the chi-square distribution [12]. The interval was defined as the set of values that satisfy the inequality $2\left(L\left(Y|\theta \right)-L\left(Y|\theta_{best}\right)\right)<\chi_{1,\;1-\propto }^{2}$, where $L\left(Y|\theta_{best}\right)$ represents the log-likelihood corresponding to the maximum likelihood estimate of θ and $\chi_{1,\;1-\propto }^{2}$ is the $\chi^{2}$ quantity considering only one degree of freedom at the level of 1−α. Thus, the confidence interval at 95% of the value θ covers all values that are twice the difference between the log-likelihood of a given value of θ and the log likelihood of the best estimate of a θ that is less than 3.84.

### 2.6. Software Application

Parameter searches guided by the objective function were carried out with Excel’s Solver tool. Excel is a program that is part of the Microsoft Office suite. Solver operates on a set of cells known as decision variable cells, which are used to compute the formulas in both the objective and constraint cells. It iteratively adjusts the values in these variable cells to satisfy the constraints and achieve the desired outcome in the objective cell.

## 3. Results

**Figure 1:**
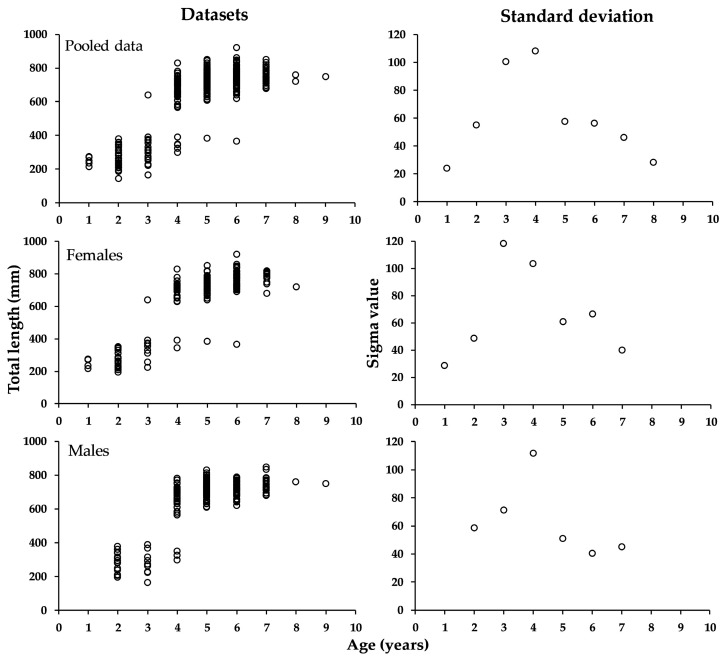
Length-at-age data and the observed variability at age for the three data sources.

In the three datasets of total length (Figure 1, first column), the curve might be asymptotic but is more likely to be sigmoid. The standard deviation (Figure 1, second column) displays a bell shape, increasing and then decreasing. For this reason, it is possible to parametrize the model with constant, depensatory, or compensatory variance. Note that in the pooled data, two values are obtained for an age of 8 years, and for this reason, these are represented in the graph of sigma value. This is not the case for the data separated by sex, because only one record was observed at ages of 8 (both sexes) and 9 years (males).

### 3.1. Schnute Growth Model

When fitting the Schnute growth model, the lowest BIC was calculated with sample variance, here called the observed variance (Table 1). The second lowest BIC was observed for the constant variance, followed by the multiplicative error structure. This result was the same for the three data sources: pooled data, females, and males. However, in females, 6% of the evidence is in favor of the additive error structure.

**Figure 2:**
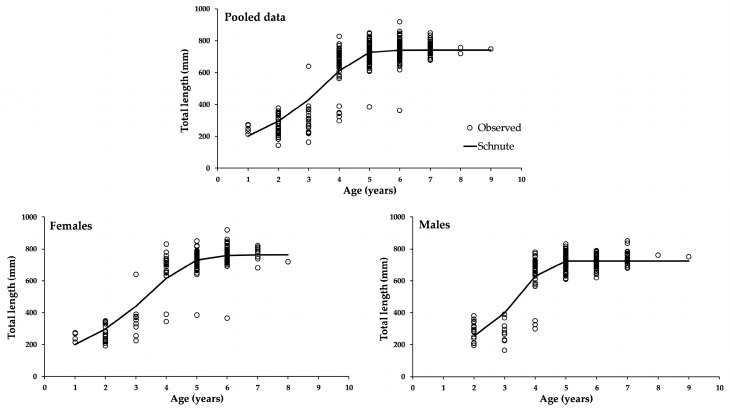
Growth curves of the Schnute model fitted with the observed variance for the three data sources. Lines represent a sigmoid asymptotic curve.

The growth patterns observed for (*Cynoscion othonopterus*) were best represented by the Schnute model with observed variance, with a BIC weighting of 100% (Table 1), showing that the other curves had no support for the data. The parameters of the Schnute model version 1 were selected using the three criteria (observed, additive, and multiplicative) and described a sigmoidal curve that did not extrapolate back to the time axis. Instead, this curve had the time axis as the lower asymptote. This curve shape was inferred because the parameters met the principles –b*ln(Y_2_/Y_1_)/(τ*_2_-*τ*_1_) < a* and *b ≤* 0. The parameters obtained once the equation was used to transform data into *L_∞_*, *k*, or t* were *L_∞_* = 741.2, *k* = 3.7 y^−1^, and t* = 3.76 years. As the best model, it obtained over 90% of the Bayesian weight. Figure 2 shows only these three curves for the three data sources, and the parameters are shown in (Table 2). For pooled data, the converted parameters from Schnute into von Bertalanffy were *L_∞_* = 935.9, *k* = 0.275 y^−1^, and *t*_0_ = 0.244. On the other hand, the version representing the logistic model gave the following parameters: *L_∞_* = 783.8, *k* = 0.857 y^−1^, and t* = 2.48 years.

### 3.2. Logistic Growth Model

**Figure 3:**
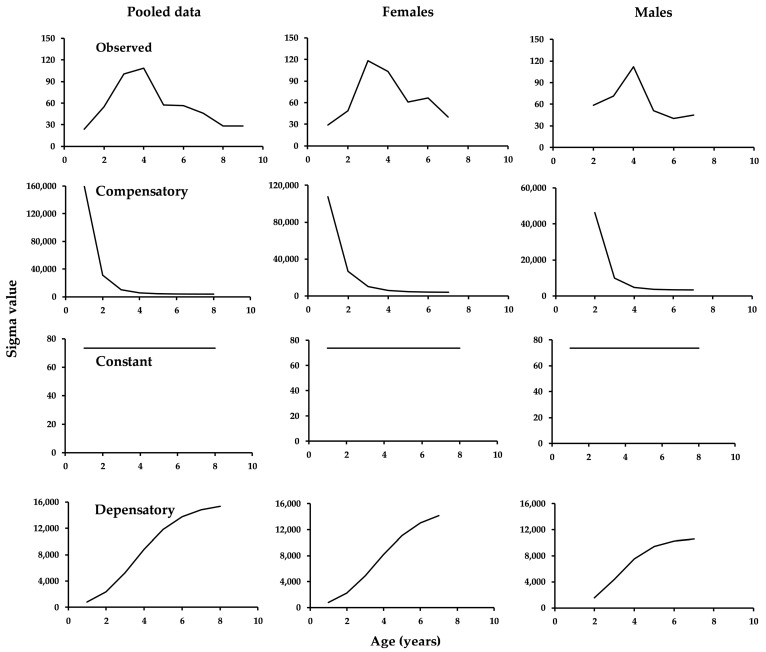
Variability at age of the four criteria used for the three data sources.

Testing the logistic growth models using the four variance criteria revealed that the model with observed variance yielded the lowest BIC (Table 3), followed by the compensatory variance in pooled data and males, but in females, the constant variance ranked second. Depensatory variance was ranked in last place for the three data sources (Table 3). Figure 3 presents the variance trajectories based on observed, constant, depensatory, and compensatory criteria for the total length data.

**Figure 4:**
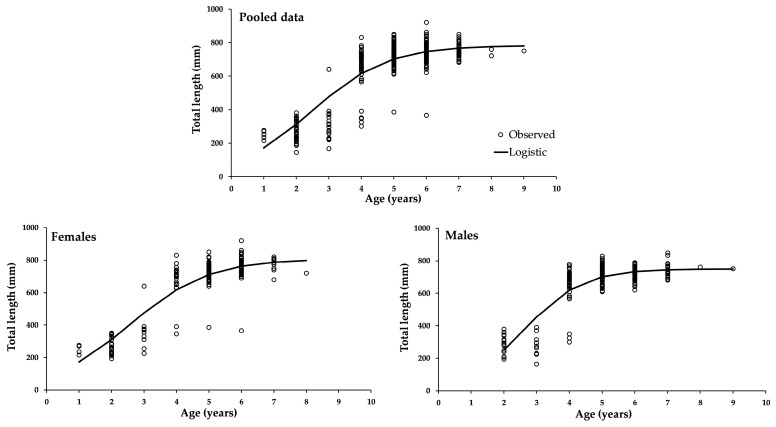
Growth curves of the logistic model fitted with the observed variance for the three data sources. Lines represent a sigmoid asymptotic curve.

Since the logistic model fitted with observed variance provided the best fit, its parameters are presented in Table 4. The curve trajectories for the three data sources are shown in Figure 4. There are significant differences between males and females in the parameters *L_∞_* and *k*. However, no significant difference was observed in the inflection point. According to the upper limit of the 95% confidence interval, females can grow to over 817 mm, while males reach a maximum of approximately 757 mm. Males exhibit a significant higher growth rate than females.

## 4. Discussion

The significant variability in length at age in fish could reflect high environmental variability and possibly competition for food. This variability remains significant as organisms age. Earlier papers on the individual growth evaluation of wild fishes assumed that constant variation would be most appropriate to use, but when a multi-criteria approach was used, the observed variation was the most appropriate according to the BIC [9]. Most growth studies for wild or farmed animals assume constant variation at an identifiable age. The advantage of using observed variance is the recognition of the intrinsic variability in length at age, which is not discernable when the objective function is solved using the conventional constant-variance assumption or a monotonically increasing or decreasing approach.

Individual variability by age should be considered to parameterize models and obtain better results [7]. This approach has been used for both wild [13] and farmed fish [9], concluding that this should be a common practice. Assessing sigma values by optimizing observed variability led to improved analysis of length-at-age data. If the variance is assumed to be constant using the conventional method, the improved analysis of length-at-age data cannot be documented. The observed variance could demonstrate an inherent variability of length-at-age data. In this study, the BIC made evident the differences between the various variance criteria for the species studied. In other words, having confidence in the traditional criteria (constant variance) could lead to the conclusion that the error structure is irrelevant. However, fitting the growth model with observed variance allowed us to show here that this approach produces robust results; that is, the observed variance produced the most plausible fits.

BIC quantifies the relative likelihood of each model being the best approximation of the true biological process that generated the data. BIC weight (*Wi*) represents the probability that model *i* is the best model (given the data and assuming the true model is in the candidate set). In growth analysis, models represent different hypotheses about growth. BIC weights help to assess which hypothesis is most supported by the data, while penalizing model complexity. The BIC balances model fit (likelihood) with parsimony (penalizing more complex models). This normalization allows *Wi* to be interpreted like probability (a higher *Wi* means stronger evidence that the model best explains data). In essence, BIC weights provide a way to rank competing biological hypotheses with a quantifiable measure of support, grounded in statistical theory.

Previous studies on the growth of Gulf corvina (*C. othonopterus*) have applied the von Bertalanffy growth model (VBGM) without considering alternative models. In the present study, two growth models were fitted. The result here is an asymptotically sigmoid shape as the best pattern to describe the growth of Gulf corvina. However, [14] found biphasic growth to be the best pattern. This biphasic growth pattern is affected by reproductive age, and this must be included in fitting a growth model [15]. Gherard et al. [2] reported that a shift in energy allocation occurs in Gulf corvina, supporting the biphasic growth mentioned by [14]. The VBGM has been modified as an approach to fitting a biphasic growth pattern in fishes [15,16]. Erisman [17] indicated that biphasic growth could be appropriate for Gulf corvina but avoided its use because of the difficulty of applying further fishery analysis. Previously, [15] showed how to describe the growth performance of a species: the best model must be applied independently if the species is a fishery resource. In the present study, a sigmoid curve was the most accurate growth pattern established by the Schnute model. The versatility of the Schnute model allowed us to determine that the model can generate the curve that best fits the data, namely a sigmoid curve, also known as case 1 of the Schnute model. Of course, this depends on the combined values of *a* and *b* in the dataset used for the present study.

As mentioned above, previous studies on Gulf corvina growth have produced different results because of the models that were used. Bolser et al. [18] decided to use the Schnute model, establishing the maximum length at an age of 8 years and the minimum length at an age of 1 year, and only estimated the model parameters *a* and *b*, when *Y_1_* and *Y_2_* should also have been estimated. This can be seen in the original article ([6]: page 1130), which states that four parameters must be found and only *T_1_* and *T_2_* are fixed. Graphs of the models in Table 1 of [18], which are those they report as adjusted to the original data, do not match their graphs. Therefore, it is concluded that this model was not well adjusted. Accepting then that their real models are those in Table 1 of Bolser et al. [18], while the adequately estimated parameters are found for the von Bertalanffy, logistic, and Schnute and Richards models, it can be observed that the Schnute and Richards model practically reaches an asymptote of 730 mm from an age of 5 years onwards, while with the logistic and von Bertalanffy models, the curve continues to ascend. This indicates that the Schnute and Richards model infers smaller sizes than the von Bertalanffy model at ages of 6 years and older. In this case, assuming that it was adequately adjusted via maximum likelihood, the fact that the Schnute and Richards model ranked in first place must have been due to the large amount of data from fish aged under 6 years, for which the model fits better, while these data were missing for fish aged 6 to 8 years. In this case, Bloser’s observation that the von Bertalanffy model is a better model would be correct, although statistically this is not the case for the original data. The above reinforces the ideas of Bolser et al. [18]: “The existence of discrepancies between the previous Gulf corvina growth studies and the importance of the age length relationship to the stock assessment of the fishery merit further investigation on the growth pattern of the species”. In the present study, we rethink the individual growth analysis of the Gulf corvina and conclude that an asymptotic, sigmoid-shaped curve best represents the growth pattern of this species. It is also significant to mention that Arzola-Sotelo [19] applied a multi-model approach that included the von Bertalanffy, Gompertz, logistic, and Schnute (case 1) models. Arzola-Sotelo [19] found that the logistic model yielded a *W_i_* = 99.92% (Akaike weight), while reported a *L_∞_* = 772.2 mm Lt, CI (769.4–774.9) for pooled data (male and females); these values are significantly smaller than those found in the present study (*L_∞_* = 783.8 (777.3–789.6)).

In the above studies using multi-model approaches, the von Bertalanffy model is always compared with other models. Flinn and Midway [20] suggested improving growth equations to achieve common sense in fish growth analysis. Even though in many important species in commercial fisheries, models other than the von Bertalanffy growth model have a better fit, the VBGM is better for stock assessments, mainly because it is derived from an ingrained paradigm coined in 1957 by Beverton and Holt in their book on fishery assessments [4]. It is worth mentioning that the elasmobranch group (also known as cartilaginous fish) can be contradictory in terms of results using models other than the VBGM [21,22]. On the one hand, [21] found that, for a species of skate in the family Rajidae, the logistic model was the best compared to the von Bertalanffy and Gompertz models according to the Akaike information criterion (AIC) for three datasets. On the other hand, [22] compared the Gompertz model with the von Bertalanffy model for a species belonging to the family Rhinobatidae, in which case the former was shown to be best by the AIC. The generalized use of the VBGM can also be observed in studies analyzing growth in other very important sciaenid fish in the Upper Gulf of California, as it is the most commonly used model for analyzing growth curves [23,24,25,26,27,28].

As the Gulf corvina remains the most important fishery resource in the Upper Gulf of California zone and the Colorado River Delta biosphere reserve [29], good fishery administration is required, while a detailed understanding of stock assessment and population dynamics is also needed. Individual growth parameters also need to be assessed. It is worth noting that the purpose of this study is to obtain the best parameters using novel approaches, including the observed error structure.

## 5. Conclusions

In order to parametrize individual growth models (the Schnute and logistic models), in the present study, the sample variance was found to be most effective using length-at-age data for Gulf corvina (*C. othonopterus*) from the Colorado River Delta biosphere reserve in the fishery zone. The BIC indicated that the observed error structure produced the most reasonable inferences. We conclude that the observed error structure should be explored when robust estimations of individual growth parameters are required.

## Figures and Tables

**Table 1:** The Bayesian information criterion (BIC) for the Schnute model and the three error structures. Δ_i_ is the difference and the BIC minimum value and the i value. W_i_ is the percentage of evidence in favor of model i.

Data Source	Structure	BIC	Δ_i_	W_i_ (%)
	Observed	5228	0	100
Pooled data	Additive	5291	62	0
	Multiplicative	5602	374	0
	Observed	2358	0	94
Females	Additive	2364	6	6
	Multiplicative	2480	122	0
	Observed	2574	0	100
Males	Additive	2644	74	0
	Multiplicative	2799	225	0

**Table 2:** Estimated growth parameters of (*Cynoscion othonopterus*) for each data source using the Schnute model version 1. The confidence intervals at 95% are in parentheses.

Data Source	Y_1_	Y_2_	*a*	*b*
Pooled data	203.2 (198–209)	741.2 (735–747)	3.070 (2.969–3.186)	−8.162 (−7.84–8.47)
Females	199.8 (191–209)	762.1 (751–773)	2.073 (1.985–2.180)	−5.178 (−4.86–5.47)
Males	253.7 (243–265)	724.4 (719–731)	10.813 (10.14–11.48)	−23.769 (−22.37–25.37)

**Table 3:** The Bayesian information criterion (BIC) for the logistic model and the four variance criteria. Δ_i_ is the difference between the BIC minimum value and the i value. W_i_ is the percentage of evidence in favor of model i.

Data Source	Criteria	BIC	Δ_i_	W_i_ (%)
Pooled data	Observed	5333	0	99.38
	Compensatory	5343	10	0.61
	Constant	5351	18	0.01
	Depensatory	5566	233	0.00
Females	Observed	2381	0	99.91
	Constant	2395	14	0.09
	Compensatory	2403	23	0.00
	Depensatory	2478	98	0.00
Males	Observed	2602	0	100
	Compensatory	2647	45	0
	Constant	2675	73	0
	Depensatory	2760	158	0

**Table 4:** Parameter estimates and their 95% confidence intervals (CIs) for the logistic growth model with the observed error structure. In this model, *L_∞_* denotes the asymptotic length, *t^⁎^* represents the inflection point of the sigmoid curve, and *k* is the growth coefficient.

Parameter	Data Source	Optimum (CI)
*L∞* (mm)	Pooled data	783.8 (777.3–789.6)
	Females	806.7 (796.7–816.7)
	Males	749.5 (742.6–756.4)
*k* (years^−1^)	Pooled data	0.857 (0.823–0.893)
	Females	0.829 (0.785–0.881)
	Males	1.126 (1.053–1.209)
t* (years)	Pooled data	2.48 (2.42–2.54)
	Females	2.57 (2.48–2.66)
	Males	2.62 (2.52–2.71)

## Data Availability

Data are available from the CONABIO Database SNIB-CONABIO; http://www.conabio.gob.mx/institucion/cgi-bin/datos.cgi?Letras=L&Numero=298 (accessed on 28 February 2025).

## Abbreviations

The following abbreviations are used in this manuscript:

AIC	Akaike information criterion
BIC	Bayesian information criterion
CI	Confidence interval
CIBNOR	Centro de Investigaciones Biológicas del Noroeste
CONABIO	Comisión Nacional para el Conocimiento y Uso de la Biodiversidad
CONAHCYT	Consejo Nacional de Humanidades, Ciencias y Tecnologías
SECIHTI	Secretaría de Ciencias Humanidades Tecnología e Innovación
SNIB	Sistema Nacional de Información sobre biodiversidad
SNII	Sistema Nacional de Investigadoras e Investigadores
VBGM	von Bertalanffy growth model
